# Trans-arcuate line vs. transumbilical access in single-port TEP groin hernia repair: a propensity score-matched retrospective cohort study

**DOI:** 10.3389/fsurg.2026.1898654

**Published:** 2026-10-13

**Authors:** Ji-Feng Zhang, Chun-Tu Wen, Ning-Shi Zhang, Gan-Kun Wang, Shun-Kang Zheng, Pei-De Liang, Xiao-Qian Luo, Qi-Yan Li, Shang-Jun Zhou, Jun Liu

**Affiliations:** 1 Department of Surgery, Dongguan Hospital of Guangzhou University of Chinese Medicine, Dongguan, China; 2 Department of Laboratory Medicine, Dongguan Hospital of Guangzhou University of Chinese Medicine, Dongguan, China

**Keywords:** groin hernia, TEP, trans-arcuate line, transumbilical, VAS

## Abstract

**Background/objective:**

Groin hernia is a common surgical condition requiring improved techniques. This study compares perioperative and postoperative outcomes of trans-arcuate line vs. pre-posterior sheath transumbilical approaches in single-port totally extraperitoneal (TEP)hernia repair.

**Method:**

A retrospective cohort study included 297 patients with groin hernia who underwent single-port TEP hernia repair. After excluding 5 cases and applying propensity score matching (PSM), patients were divided into the transumbilical group (*n* = 83) and the trans-arcuate line group (*n* = 83) according to the surgical approach. Outcomes included operative time, peritoneal injury rate, postoperative pain score measured by the Visual Analogue Scale (VAS), haematoma, and intraoperative blood loss. CUSUM analysis was performed to assess operative-time learning curves in both groups, followed by subgroup analyses stratified into the learning and post-transition phases. Baseline characteristics were generally comparable between groups in the learning phase, although some imbalances were observed given the small sample size. For post-transition cases, propensity score re-matching was performed before comparison.

**Results:**

After PSM, the trans-arcuate line group had significantly shorter operative time and a markedly lower incidence of intraoperative peritoneal injury than the transumbilical group. Early postoperative VAS pain scores were also significantly lower in the trans-arcuate line group (*P* < 0.001). However, no statistically significant differences were observed in intraoperative blood loss or haematoma between the two groups. CUSUM analysis further revealed a longer learning phase in the trans-arcuate line group. Within the learning phase, this group had a lower peritoneum or hernia sac tearing rate, whereas in the post-transition phase, it had shorter operative time. Notably, when the comparison was restricted to post-transition cases after re-matching, peritoneum or hernia sac tearing rate and VAS scores no longer differed between groups.

**Conclusion:**

After PSM and phase-stratified analysis, the trans-arcuate line approach was associated with a lower peritoneum or hernia sac tearing rate during the learning phase, and with shorter operative time after proficiency was reached. These findings suggest that the trans-arcuate line approach may offer distinct advantages at different stages of the learning curve, and the trans-arcuate line approach may be considered a favorable alternative after sufficient surgical experience has been acquired.

## Introduction

Groin hernia represents a prevalent condition frequently observed in surgical practice, exhibiting a notably high incidence in male patients (1). This condition not only induces pain and discomfort, thereby significantly impairing patients' quality of life, but it also poses the risk of severe complications, including incarceration and strangulation. As a result, groin hernia imposes a substantial disease burden and necessitates considerable healthcare resources from both patients and society (2–4). Therefore, further optimization of surgical techniques, enhancement of treatment outcomes, reduction of complications, and acceleration of recovery processes are of clear clinical importance and societal relevance.

With the widespread adoption of minimally invasive surgical concepts and techniques, laparoscopic hernia repair has emerged as a primary method for treating groin hernias. Its benefits, including reduced postoperative pain, accelerated recovery, and shorter hospital stays, are well-documented. Among these techniques, totally extraperitoneal (TEP) hernia repair, which is performed entirely outside the peritoneal cavity and minimizes interference with intra-abdominal organs, is considered to offer greater minimally invasive potential (5, 6). Currently, laparoscopic TEP surgery is advancing towards more minimally invasive single-port approaches. However, there is a lack of high-quality evidence regarding the optimal entry route for single-port TEP—such as the trans-arcuate line approach vs. the transumbilical approach—and their effects on key outcome measures, including surgical operability, intraoperative visualization, preservation of peritoneal integrity, and postoperative pain management. This topic remains a subject of academic debate (7–9). Several existing studies propose that single-port TEP might provide enhanced postoperative pain management and greater patient satisfaction in comparison to traditional multi-port techniques. However, definitive conclusions regarding the comparative advantages of various entry techniques remain elusive (10). For example, the trans-arcuate approach and the transumbilical approach may differ in terms of technical complexity in establishing an extraperitoneal working space, potential risks of peritoneal injury, and postoperative pain perception. These differences could have direct implications on surgical efficiency and the quality of patients' short-term recovery. Unfortunately, most current studies are limited by small sample sizes and lack systematic comparative analysis.

To address this clinical issue, this retrospective cohort study will systematically collect and analyse electronic medical records of groin hernia patients undergoing single-port TEP surgery, focusing on comparing the trans-arcuate line and transumbilical approaches. We compared the groups with respect to operative duration, intraoperative peritoneal breach incidence, intraoperative blood loss, postoperative pain scores and the incidence of postoperative haematoma. This aims to provide objective, scientific evidence for optimising single-port TEP approach selection in clinical practice.

## Materials and methods

### Study population

This study included groin hernia patients treated at Dongguan Hospital of Guangzhou University of Chinese Medicine between January 2021 and December 2025 (Figure 1). All diagnoses complied with the clinical diagnostic criteria outlined in the “Guidelines for the Diagnosis and Treatment of Inguinal Hernia”. Inclusion Criteria: Patients undergoing TEP were selected, with surgical approaches restricted to trans-arcuate or transumbilical routes. Exclusion criteria: concurrent abdominal surgery; postoperative loss to follow-up; incomplete clinical records. Participants were divided into two groups based on the surgical approach used: the trans-arcuate line group and the transumbilical group. All procedures were performed by the same surgical team, led by a consultant surgeon (J.-F.Z.) with over 20 years' experience in hernia surgery and extensive expertise in laparoscopic groin hernia repair. J.-F.Z. acted as chief surgeon in every case included in this study; the other named authors assisted, principally by controlling the laparoscope. Each operation involved one chief surgeon and one assistant, and all assistants were junior physicians with more than one year of experience in laparoscopic surgery. Because a single surgeon performed every procedure, variation between surgeons was eliminated; conversely, that surgeon's cumulative experience increased in parallel with the change of technique, a point taken up in the Limitations.

**Figure 1:**
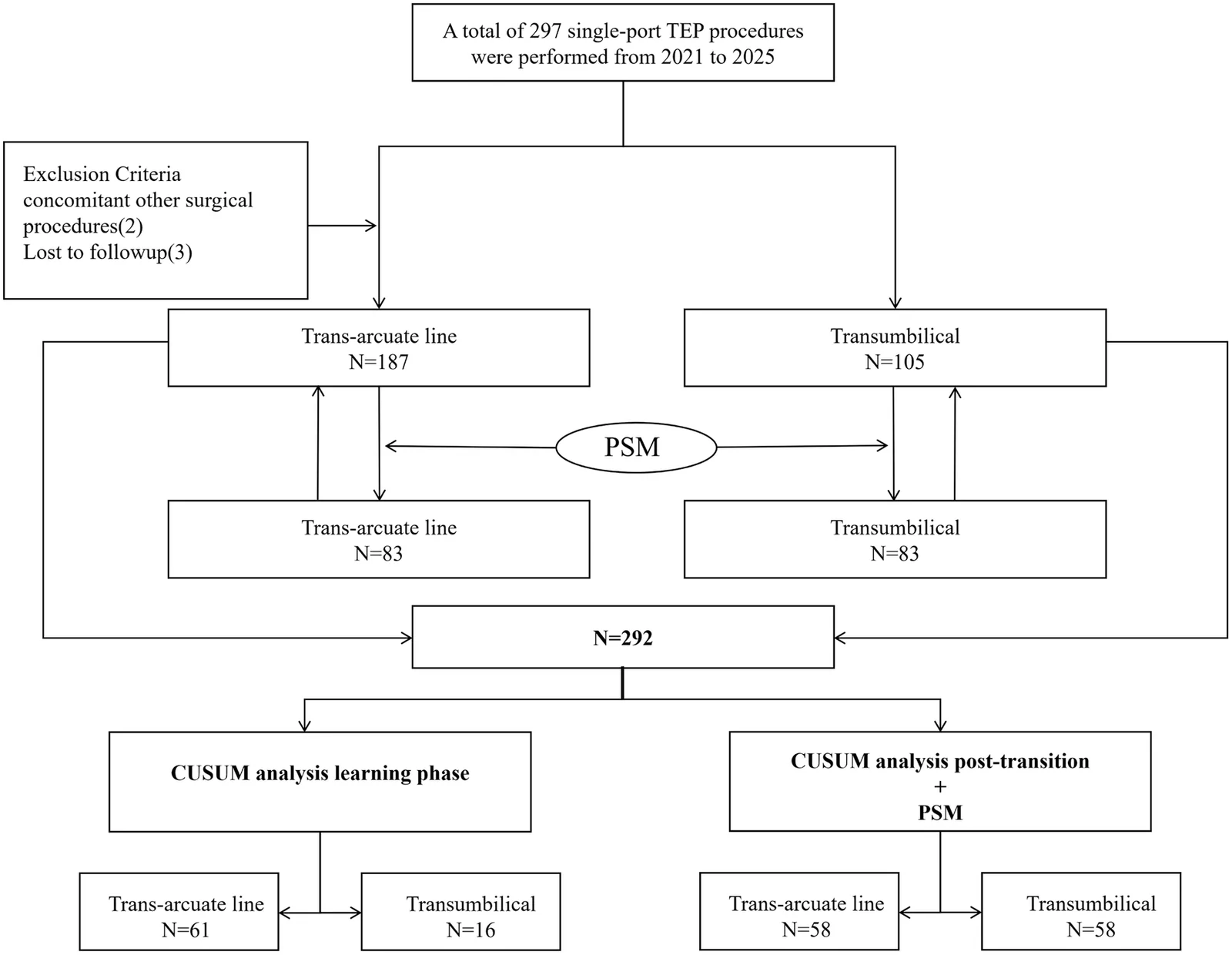
The schematic representation of case screening for each group.

### Trans-arcuate approach

The patient was positioned supine with head-down, feet-up at 15°. Both surgeon and assistant stood on the patient's unaffected side; in bilateral hernias, both stood opposite the operative side. Incision site: A 2 cm midline longitudinal incision 4 cm below the umbilicus. Surgical procedure: Incision through skin and subcutaneous tissue to expose the anterior sheath of the rectus abdominis muscle. Make a 2 cm longitudinal incision in the anterior sheath 1 cm lateral to the midline. Blunt dissection of the rectus abdominis muscle to enter the retro-rectus space. Using the right index finger, perform lateral blunt dissection between the rectus abdominis muscle and its posterior sheath to enlarge the operative field. The rectus abdominis muscle is then retracted laterally. A single-port trocar is inserted and CO_2_ gas is introduced, maintaining pressure at 12 mmHg. A 30°laparoscope is inserted to visualise the arcuate line and adjacent anatomy. The arcuate line is found to be predominantly located directly beneath the incision (4–5 cm below the umbilicus) or slightly more distally. Incision at the junction of the arcuate line and transverse abdominal fascia provided entry into the preperitoneal space, extending caudally to reach the Retzius space. Dissection is performed under direct vision to enter the space of Retzius (the retropubic space), exposing the pubic symphysis and Cooper's ligament. The inferior epigastric vessels are identified and preserved. Lateral to these vessels, the space of Bogros (the lateral iliac fossa/retroperitoneal space) is developed. The myopectineal orifice (Fruchaud's triangle) is then dissected. After opening the internal spermatic fascia, the hernia sac is carefully dissected—first laterally, then medially, and finally at its base—before being reduced. In cases of a scrotal or large hernia sac, it is transected in the mid-portion and the proximal end is ligated. Deperitoneate the spermatic cord to 6 cm. Dissect the vas deferens to the junction of the obturator urachal artery. Create a sufficiently large preperitoneal space to accommodate the mesh. Position the mesh, ensuring it is fully unfolded and completely covers the obturator foramen. A self-gripping polyester mesh (ProGrip; 15 cm × 9 cm) was deployed endoscopically over the inguinal region. No additional fixation—neither sutures, tacks nor surgical adhesive—was used, fixation relying entirely on the self-gripping surface of the prosthesis. The same prosthesis and the same fixation policy were used in both study periods and in all 292 patients, so that no second intervention changed alongside the access route. Throughout dissection, meticulously protect the inferior epigastric vessels, spermatic vessels, and vas deferens. Finally, under direct visualisation, slowly release the CO_2_ gas, remove the single-port trocar, and suture the incision.

### Transumbilical approach (pre-posterior sheath transumbilical approach) group

Position the patient supine with head-down, feet-up at 15°. Both surgeon and assistant stand on the patient's unaffected side; for bilateral hernias, both stand opposite the operative side. Incision selection: Arc-shaped incision 2 cm long, 1 cm below the umbilical margin. Surgical procedure: Incise skin and subcutaneous tissue to expose the anterior sheath of the rectus abdominis muscle. Make a 2 cm longitudinal incision in the anterior sheath 1 cm lateral to the midline. Bluntly dissect the rectus abdominis muscle to enter the posterior rectus sheath space. Using the right index finger, bluntly dissect laterally between the rectus abdominis muscle and its posterior sheath to enlarge the operative field. The rectus abdominis muscle was then retracted laterally to insert a single-port trocar. Carbon dioxide gas was introduced, maintaining pressure at 12 mmHg. A 30° laparoscope was inserted. Dissection proceeded along the surface of the posterior rectus sheath. Dissection is carried out along the surface of the posterior rectus sheath inferiorly. Near the arcuate line, the transversalis fascia is incised to enter the preperitoneal space, which is then extended caudally to reach the space of Retzius. The remaining surgical steps, including mesh placement, are essentially the same as those in the trans-arcuate line approach.

### Indicators

We collected baseline patient data including age and gender. Additional variables comprised history of lower abdominal surgery, hernia classification, hernia side, and American Society of Anesthesiologists (ASA) anaesthetic risk score. Observed indicators included operative time (defined as the primary outcome), blood loss, intraoperative complications (peritoneal or hernial sac rupture, also defined as the primary outcome), and postoperative VAS scores (assessed using the standard 10-point VAS recommended by the International Association for the Study of Pain, where 0 points represent no pain and 10 points represent the most severe pain imaginable). The scale scores shown in the text are the original VAS scores recorded at rest 24 h after surgery, which were retrospectively extracted from medical records), postoperative complications (haematoma, confirmed by ultrasound). Recurrence and length of hospital stay were not included as study outcomes. Follow-up was not standardised for the ascertainment of recurrence: assessment combined clinical examination with ultrasonography or computed tomography according to clinical indication rather than at fixed, protocol-defined intervals, and neither the duration of follow-up nor the number of patients assessed at each visit could be established reliably for both periods, which differed in calendar time. These outcomes are therefore not reported.

### Statistical analysis

We confirm that the present study was conducted and reported in accordance with the Strengthening the reporting of observational studies in epidemiology (STROBE) guideline. A detailed STROBE checklist is provided in the Supplementary Table S1. Qualitative variables were analysed using the chi-square test or Fisher's exact test. For quantitative variables, normality was assessed; *t*-tests were employed for normally distributed samples, while the Mann–Whitney *U*-test was used for non-normally distributed data. The sample size was not determined by a formal power calculation; it was determined by the number of eligible patients treated during the study period, and no *post hoc* claim of adequate power is made. Baseline differences were addressed using propensity score matching. The nearest neighbor matching algorithm was applied with a 1:1 matching ratio, a caliper width of 0.2 standard deviations of the logit of the propensity score, and matching without replacement to ensure the independence of matched pairs. To test whether the observed differences could be attributed to the accumulation of surgical experience rather than the surgical approach itself, we analyzed the true chronological sequences comprising all 187 and 105 operations, respectively, and performed a cumulative sum (CUSUM) analysis of operative time. The CUSUM was calculated as the cumulative sum of deviations from the overall mean operative time of the sequence, plotted in the order in which the operations were performed. The calculation was based on the complete consecutive surgical series of each group rather than a matched cohort, because excluding more than half of the cases and renumbering the remaining cases would disrupt the chronological information upon which the learning curve depends. The peak of each curve was taken as the estimate of the transition point. Subgroup analyses were conducted, stratified into the learning phase and the post-transition phase. For cases in the post-transition phase, propensity score re-matching was performed before comparison.

## Results

### Baseline characteristics

A comparative analysis of baseline characteristics between the two surgical approach groups revealed no statistically significant differences in age, gender, hernia classification, or hernia distribution, indicating comparability between the patient populations. However, statistical differences were observed in ASA scores; we performed propensity score matching (PSM) and subsequently obtained 83 cases each for both approaches. After matching, no statistically significant differences were found in baseline characteristics, as presented in Tables 1, and 2. One consequence of matching must be made explicit. The trans-arcuate line group contained no patients with an ASA score of III or IV, whereas the transumbilical group contained 20 (Table 1). This is complete separation rather than imbalance, and because no counterparts existed for these patients, all 20 were excluded by matching; the matched cohort therefore comprised patients with an ASA score of I or II only (Table 2). Direct comparison of cases beyond the transition phase without re-matching would have reintroduced the imbalance in ASA classification. Therefore, propensity score matching was reapplied to these cases. After re-matching, no significant differences in baseline characteristics were observed between the two groups (Table 3), in which the ASA distribution is also presented. The baseline characteristics of the two groups in the learning phase were generally comparable. However, given that there were only 16 cases in the transumbilical group, there was a certain degree of imbalance in some variables (sex, hernia laterality, and ASA grade), so the results should be interpreted with caution (Table 4).

**Table 1:** Baseline clinical data and patient characteristics before PSM.

Variable	Total (*n* = 292)	Trans-arcuate (*n* = 187)	Transumbilical (*n* = 105)	Statistic	*P*	SMD
Age, Mean ± SD	56.45 ± 16.54	55.27 ± 17.01	58.53 ± 15.52	*t* = −1.621	0.106	0.210
Sex, *n* (%)				*χ*^2^ = 0.047	0.828	
Male	277 (94.86)	177 (94.65)	100 (95.24)			0.028
Female	15 (5.14)	10 (5.35)	5 (4.76)			−0.028
Hernia type, *n* (%)				-	0.611	
Indirect inguinal hernia	196 (67.12)	124 (66.31)	72 (68.57)			0.049
Direct inguinal hernia	55 (18.84)	39 (20.86)	16 (15.24)			−0.156
combined hernia	36 (12.33)	21 (11.23)	15 (14.29)			0.087
Femoral hernia	5 (1.71)	3 (1.60)	2 (1.90)			0.022
Hernia laterality, *n* (%)				*χ*^2^ = 2.965	0.227	
Left	111 (38.01)	69 (36.90)	42 (40.00)			0.063
Right	146 (50)	91 (48.66)	55 (52.38)			0.074
Bilateral	35 (11.99)	27 (14.44)	8 (7.62)			−0.257
History of abdominal surgery, *n* (%)				*χ*^2^ = 0.740	0.390	
No	240 (82.19)	151 (80.75)	89 (84.76)			0.112
Yes	52 (17.81)	36 (19.25)	16 (15.24)			−0.112
ASA score, *n* (%)				-	<.001	
I	224 (76.71)	162 (86.63)	62 (59.05)			−0.561
II	48 (16.44)	25 (13.37)	23 (21.90)			0.206
III	18 (6.16)	0 (0.00)	18 (17.14)			0.455
IV	2 (0.68)	0 (0.00)	2 (1.90)			0.139

**Table 2:** Baseline clinical data and patient characteristics after PSM.

Variable	Total (*n* = 166)	Trans-arcuate (*n* = 83)	Transumbilical (*n* = 83)	Statistic	*P*	SMD
Age, Mean ± SD	58.19 ± 16.15	58.69 ± 16.21	57.70 ± 16.17	*t* = 0.393	0.695	−0.061
Sex, *n* (%)				*χ*^2^ = 0.000	1.000	
Male	156 (93.98)	78 (93.98)	78 (93.98)			0.000
Female	10 (6.02)	5 (6.02)	5 (6.02)			0.000
Hernia type, *n* (%)				-	0.943	
Indirect inguinal hernia	116 (69.88)	58 (69.88)	58 (69.88)			0.000
Direct inguinal hernia	28 (16.87)	15 (18.07)	13 (15.66)			−0.066
combined hernia	18 (10.84)	8 (9.64)	10 (12.05)			0.074
Femoral hernia	4 (2.41)	2 (2.41)	2 (2.41)			0.000
Hernia laterality, *n* (%)				—	0.935	
Left	66 (39.76)	34 (40.96)	32 (38.55)			−0.050
Right	91 (54.82)	45 (54.22)	46 (55.42)			0.024
Bilateral	9 (5.42)	4 (4.82)	5 (6.02)			0.051
History of abdominal surgery, *n* (%)				*χ*^2^ = 0.195	0.659	
No	142 (85.54)	72 (86.75)	70 (84.34)			−0.066
Yes	24 (14.46)	11 (13.25)	13 (15.66)			0.066
ASA score, *n* (%)				*χ*^2^ = 0.132	0.717	
I	126 (75.9)	64 (77.11)	62 (74.70)			−0.055
II	40 (24.1)	19 (22.89)	21 (25.30)			0.055

**Table 3:** Baseline clinical data and patient characteristics of post-transition and after PSM.

Variable	Total (*n* = 116)	Trans-arcuate (*n* = 58)	Transumbilical (*n* = 58)	Statistic	*P*	SMD
Age, Mean ± SD	58.22 ± 16.52	58.31 ± 16.67	58.14 ± 16.52	*t* = 0.056	0.955	−0.010
Sex, *n* (%)				*χ*^2^ = 0.000	1.000	
Male	109 (93.97)	55 (94.83)	54 (93.10)			−0.068
Female	7 (6.03)	3 (5.17)	4 (6.90)			0.068
Hernia type, *n* (%)				—	0.821	
Indirect inguinal hernia	77 (66.38)	39 (67.24)	38 (65.52)			−0.036
Direct inguinal hernia	26 (22.41)	14 (24.14)	12 (20.69)			−0.085
combined hernia	11 (9.48)	4 (6.90)	7 (12.07)			0.159
Femoral hernia	2 (1.72)	1 (1.72)	1 (1.72)			0.000
Hernia laterality, *n* (%)				-	1.000	
Left	39 (33.62)	20 (34.48)	19 (32.76)			−0.037
Right	69 (59.48)	34 (58.62)	35 (60.34)			0.035
Bilateral	8 (6.9)	4 (6.90)	4 (6.90)			0.000
History of abdominal surgery, *n* (%)				*χ*^2^ = 0.058	0.809	
No	95 (81.9)	48 (82.76)	47 (81.03)			−0.044
Yes	21 (18.1)	10 (17.24)	11 (18.97)			0.044
ASA score, *n* (%)				*χ*^2^ = 0.000	1.000	
I	94 (81.03)	47 (81.03)	47 (81.03)			0.000
II	22 (18.97)	11 (18.97)	11 (18.97)			0.000

**Table 4:** Baseline clinical data and patient characteristics of learning phase.

Variable	Total (*n* = 77)	Trans-arcuate (*n* = 61)	Transumbilical (*n* = 16)	Statistic	*P*	SMD
Age, Mean ± SD	56.65 ± 16.23	56.46 ± 16.42	57.38 ± 15.96	*t* = −0.200	0.842	0.057
Sex, *n* (%)				*χ*^2^ = 0.612	0.434	
Male	71 (92.21)	55 (90.16)	16 (100.00)			0.371
Female	6 (7.79)	6 (9.84)	0 (0.00)			−0.371
Hernia type, *n* (%)				—	0.930	
Indirect inguinal hernia	51 (66.23)	39 (63.93)	12 (75.00)			0.256
Direct inguinal hernia	14 (18.18)	12 (19.67)	2 (12.50)			−0.217
combined hernia	11 (14.29)	9 (14.75)	2 (12.50)			−0.068
Femoral hernia	1 (1.3)	1 (1.64)	0 (0.00)			−0.145
Hernia laterality, *n* (%)				*χ*^2^ = 2.252	0.324	
Left	27 (35.06)	19 (31.15)	8 (50.00)			0.377
Right	40 (51.95)	33 (54.10)	7 (43.75)			−0.209
Bilateral	10 (12.99)	9 (14.75)	1 (6.25)			−0.351
History of abdominal surgery, *n* (%)				*χ*^2^ = 0.000	1.000	
No	66 (85.71)	52 (85.25)	14 (87.50)			0.068
Yes	11 (14.29)	9 (14.75)	2 (12.50)			−0.068
ASA score, *n* (%)				*χ*^2^ = 0.326	0.568	
I	61 (79.22)	47 (77.05)	14 (87.50)			0.316
II	16 (20.78)	14 (22.95)	2 (12.50)			−0.316

### Trans-arcuate approach

Regarding the trans-arcuate surgical characteristics, the primary feature of the single-port TEP approach via the trans-arcuate route is its precise incision design and entry pathway. A midline longitudinal incision was made 4 cm inferior to the umbilicus (Figure 2A). This location is strategically chosen due to its proximity to the anatomical structure of the posterior sheath of the rectus abdominis muscle (the arcuate line), which acts as a direct “gateway” to the preperitoneal space. Following the incision and dissection of the anterior rectus sheath and muscle fibers, the posterior rectus space is accessed. A single-port trocar is then inserted, and CO_2_ pneumoperitoneum is maintained at a pressure of 12 mmHg (Figure 2B). Under direct laparoscopic visualization, the arcuate line structure beneath the incision is distinctly visible (Figures 2C,D). Making an incision at this junction facilitates direct access to the preperitoneal space, enabling caudal dissection towards the Retzius space. A significant technical advantage of this approach is the capacity to partially extract transversely transected hernial sacs or perforated peritoneum through the adjacent incision for direct visual suturing (Figure 2E). This technique presents challenges in single-port laparoscopy, whereas the trans-arcuate line pathway provides a unique convenience not achievable through other approaches. This surgical approach remains a truly single-port TEP repair, with all critical steps, including spermatic cord parietalization, suture and ligation of the pseudohernia sac, and mesh placement, performed entirely under laparoscopic visualization (Figure 3A–D).

**Figure 2:**
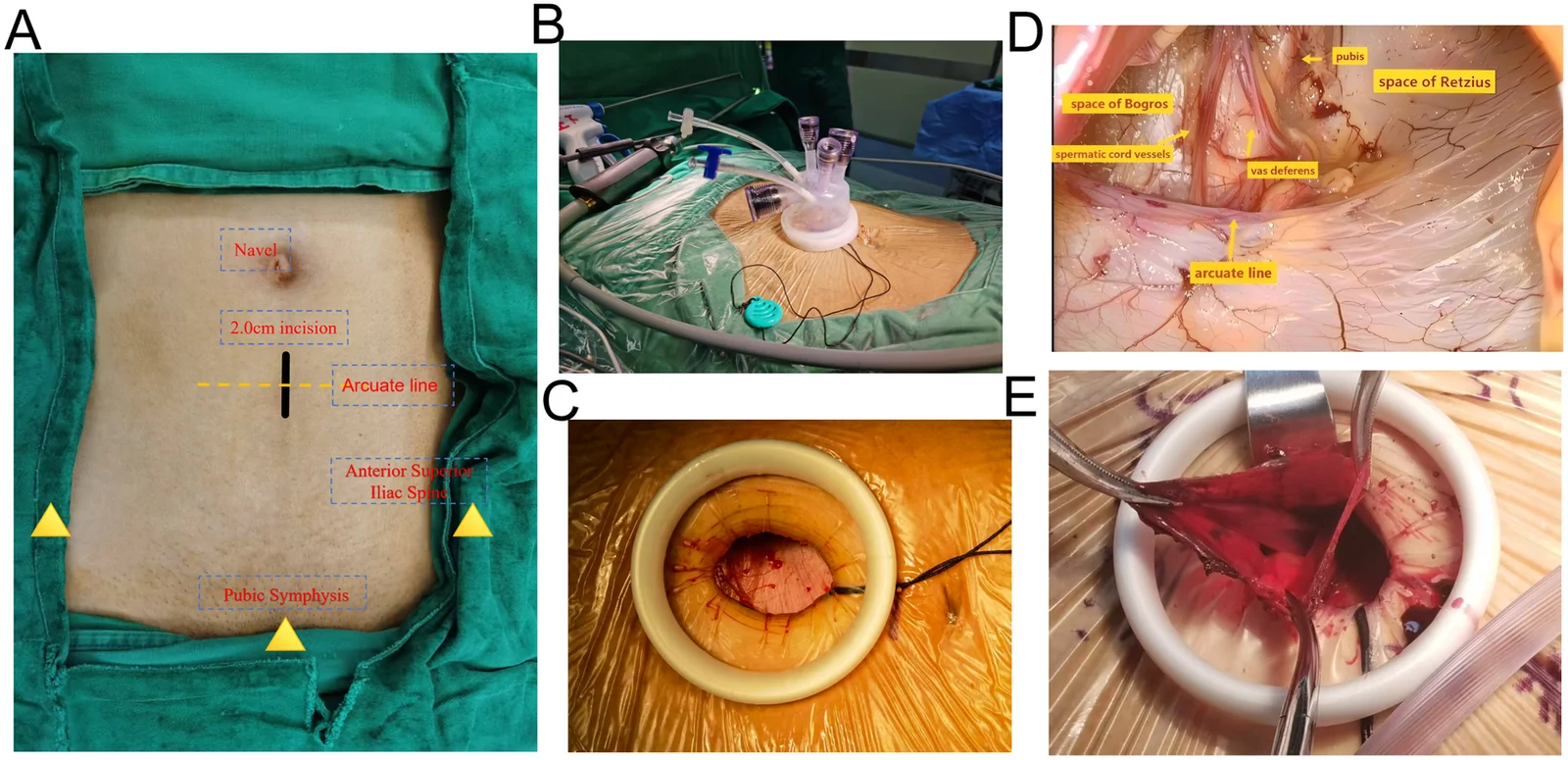
Schematic diagram of the surgical procedure. **(A)** Schematic diagram of the surgical incision. **(B)** A single hole puncture device during surgery. **(C)** Anatomical location of the arcuate line. **(D), (E)** The incision made along the arcuate line facilitates the exteriorization of the transected hernia sac, or compromised peritoneum, allowing for subsequent suturing.

**Figure 3:**
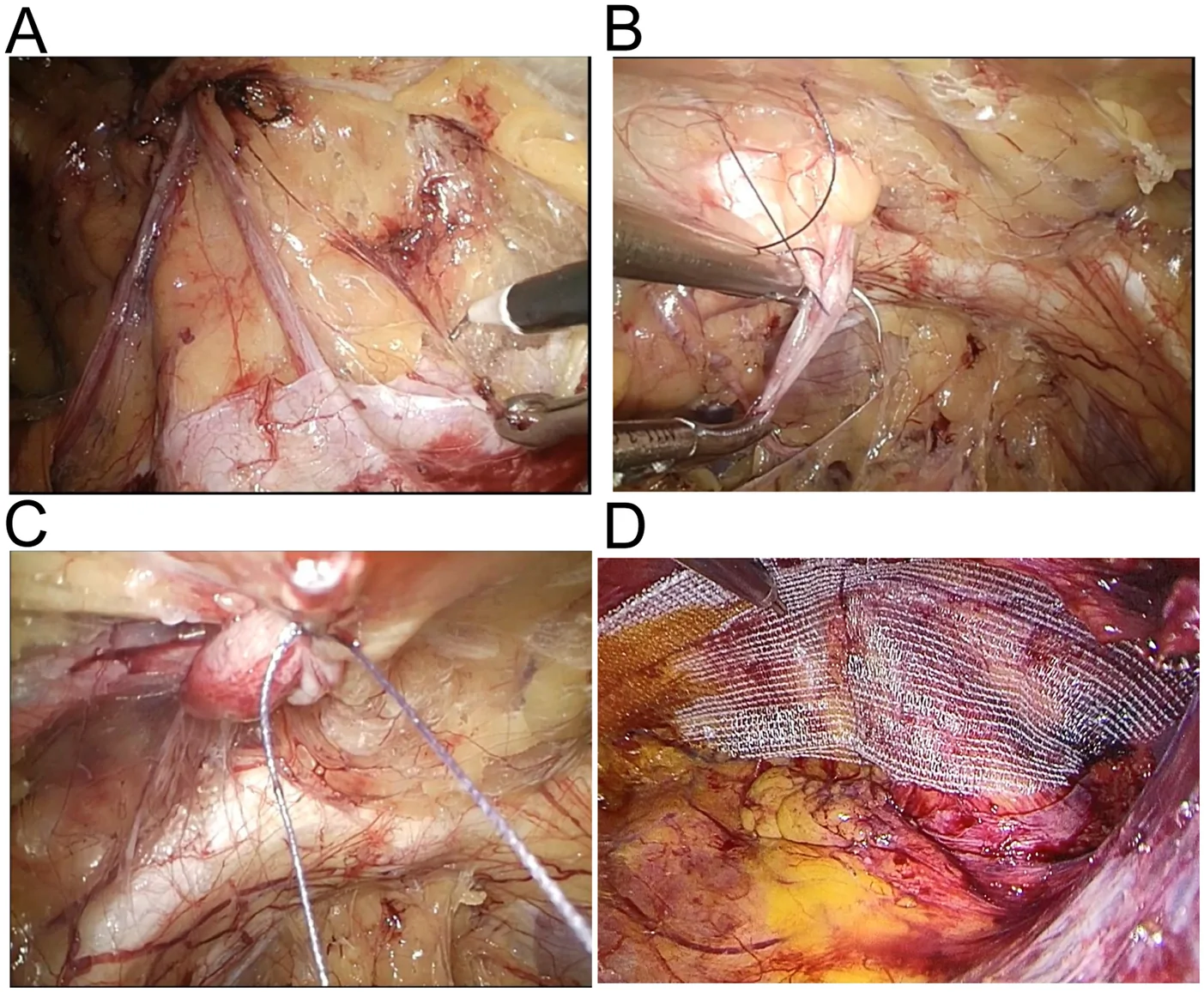
Close the defect and place the mesh under laparoscopy. **(A)** Perform blunt dissection between the spermatic cord and the posterior peritoneum under laparoscopy, typically extending distally for approximately 6–8 cm to completely separate the spermatic cord from the peritoneum. **(B)** Suture the false hernia sac of the direct hernia. **(C)** Ligate the false hernia sac of the direct hernia. **(D)** Place the mesh.

### Intraoperative and postoperative evaluation

Intraoperative assessments revealed differences between the two approaches in core efficiency and safety metrics. Firstly, operative time was significantly shorter in the trans-arcuate line approach vs. the transumbilical approach (Table 5, Figure 4A). Secondly, the peritoneum or hernia sac tearing also differed significantly between groups. The peritoneal breach incidence was 30.1% in the trans-arcuate line group, compared to 47.0% in the transumbilical group (Table 5, Figure 4B). Furthermore, there was no statistically significant difference in intraoperative blood loss between groups (Table 5). Postoperative evaluations revealed differences in early recovery between groups. VAS assessments for postoperative pain demonstrated significantly lower scores in the trans-arcuate line group compared to the transumbilical group, indicating superior pain control (Table 5, Figure 4C). However, hematoma showed no significant difference between groups (Table 5). To examine whether the difference in operative time could be attributed to a learning-curve effect, we performed the CUSUM analysis described in the Methods, on the complete consecutive series of each group (105 transumbilical and 187 trans-arcuate line procedures) rather than on the matched cohort. Following CUSUM analysis, we identified the inflection point of the operative time learning curve at case 61 in the trans-arcuate line group and at case 16 in the transumbilical group. Superficially, the transumbilical group appears to have a shorter learning curve and is easier to master (Figure 5A). However, once the learning phase is surpassed, the trans-arcuate line approach confers a more consistent speed advantage (Figure 5B). To further elucidate why the median operative time was shorter in the trans-arcuate line group than in the transumbilical group, we reanalyzed cases of post-transition phase which have been re-matched. The difference in operative time remained statistically significant, indicating that after both groups had completed experience accumulation, the trans-arcuate line approach indeed conferred an operative time advantage. This can also be interpreted as the difference in operative time being attributable to the transition and proficiency phases of the learning curve. Primary outcomes were reanalyzed, including operative time, VAS score, bleeding volume, peritoneum or hernia sac tearing, and hematoma. The detailed results are presented in the Table 6. The 77 cases in the learning phase were also analyzed. Among the aforementioned outcomes, the peritoneum or hernia sac tearing rate was the only indicator showing a statistically significant difference; details are presented in Table 7. In the learning phase, the peritoneum or hernia sac tearing rate was significantly lower in the trans-arcuate line group (27.9% vs. 62.5%, *p* = 0.017). However, given the small sample size of the transumbilical group (*n* = 16) and the observed moderate imbalances in sex, hernia laterality, and ASA grade (SMD > 0.3), the possibility that these baseline differences contributed to the observed difference in peritoneal injury cannot be entirely excluded. Nevertheless, the direction of the effect was consistent and clinically meaningful, suggesting that the trans-arcuate line approach may be more forgiving during the early learning phase. In the post-transition re-matched cohort (58 cases per group), no statistically significant differences were observed in the peritoneum or hernia sac tearing rate (31.0% vs. 36.2%, *p* = 0.694) or early postoperative VAS pain scores between the two groups. In contrast, operative time remained significantly shorter in the trans-arcuate line group. After propensity score matching and phase-stratified analysis, the trans-arcuate line approach was associated with a lower peritoneum or hernia sac tearing rate during the learning phase, and with shorter operative time after proficiency was reached.

**Figure 4:**
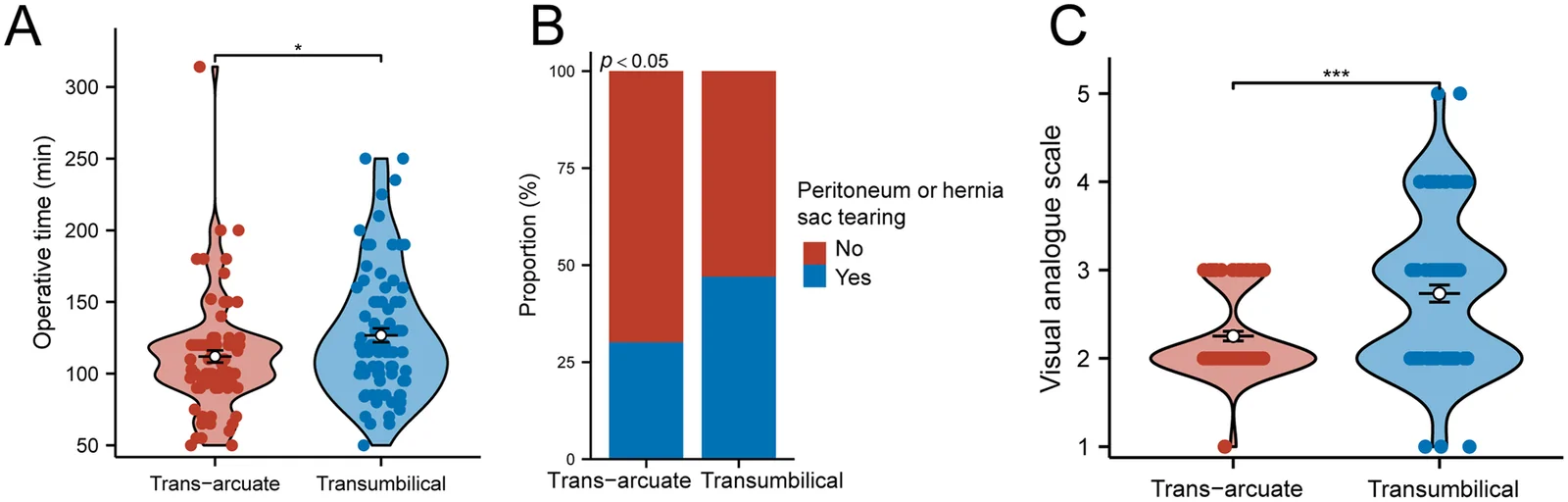
A comparative analysis of perioperative-related indices between the two groups after PSM (83vs83). **(A)** Comparison of operation time between the two groups. **(B)** Comparison of the occurrence of peritoneum or hernia sac tearing between the two groups. **(C)** Comparison of the visual analogue scale (VAS) between the two groups.

**Figure 5:**
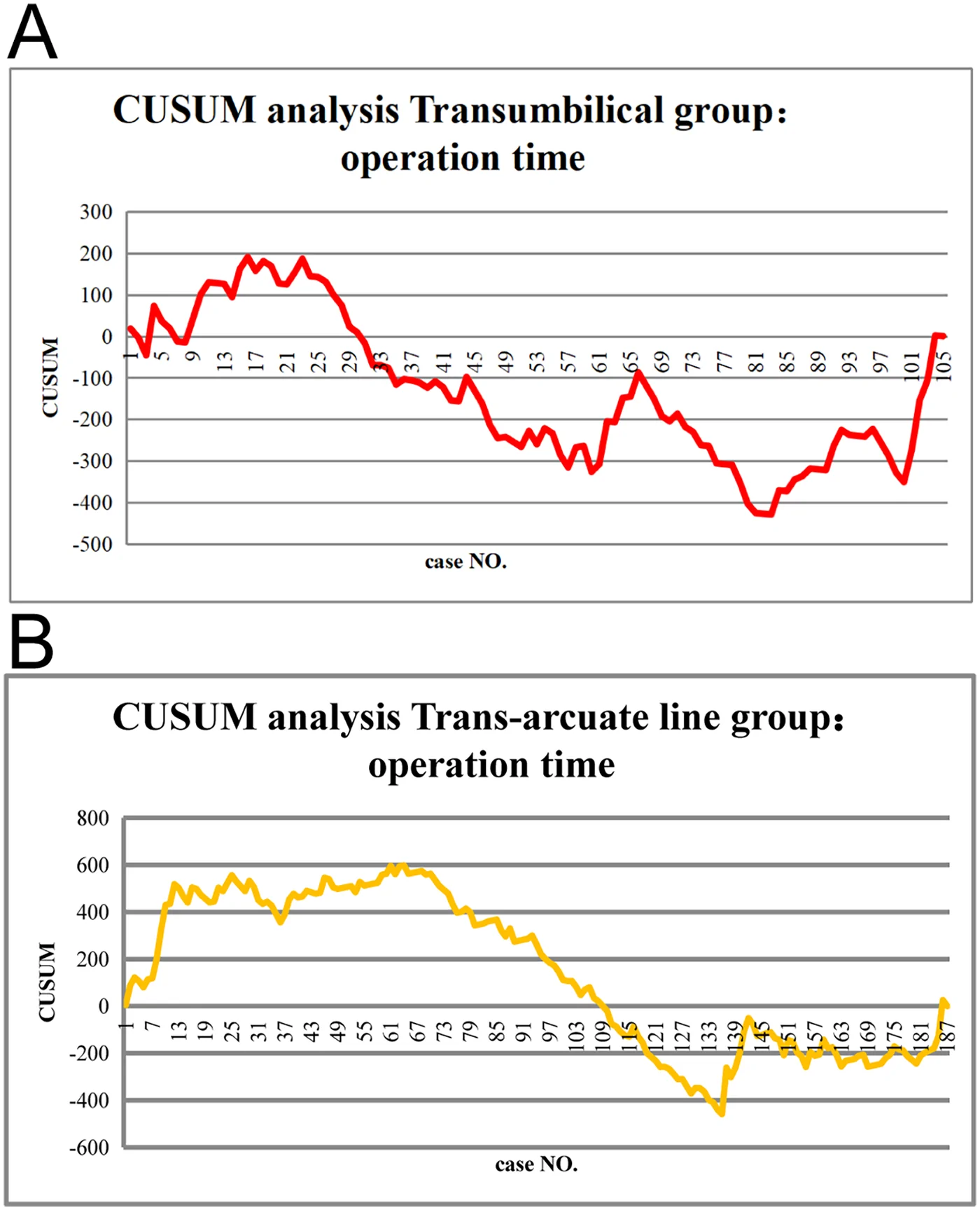
Cumulative sum (CUSUM) analysis of operative time against chronological case sequence, calculated on the complete consecutive series of each group rather than on the matched cohort. **(A)** Transumbilical group (105 consecutive cases). **(B)** Trans-arcuate line group (187 consecutive cases). The CUSUM is the cumulative sum of deviations from the overall mean operative time of the series and is standardised within each group.

**Table 5:** Analysis of key indicators in different groups.

Characteristics	Trans-arcuate (*N* = 83)	Transumbilical (*N* = 83)	*p-*value	Effect estimate (95% CI)
Operative time (min), median (IQR)	110 (93, 120)	115 (97.5, 150)	0.040	MD = −5.0 (−9.6, −0.2)
Visual analogue scale, median (IQR)	2 (2, 3)	3 (2, 3)	<0.001	MD = −1.0 (−1.3, −0.7)
Bleeding volume (mL), median (IQR)	2 (2, 5)	2 (2, 5)	0.420	MD = 0.0 (−0.5, 0.7)
Peritoneum or hernia sac tearing, *n* (%)			0.026^a^	RR = 0.64 (0.42, 0.97)
No	58 (69.9%)	44 (53.0%)		
Yes	25 (30.1%)	39 (47.0%)		
Hematoma, *n* (%)			0.367^b^	RR = 4.01 (0.46, 35.1)
No	79 (95.2%)	82 (98.8%)		
Yes	4 (4.8%)	1 (1.2%)		

a Uncorrected Pearson chi-square; ^b^Fisher's exact test; MD: difference in medians (trans-arcuate line minus transumbilical), with 95% confidence interval; RR, risk ratio (trans-arcuate line versus transumbilical), with 95% confidence interval; IQR, interquartile range.

**Table 6:** Analysis of key indicators in two groups of post-transition after PSM.

Characteristics	Trans-arcuate (*N* = 58)	Transumbilical (*N* = 58)	*p-*value	Effect estimate (95% CI)
Operative time (min), median (IQR)	104 (83,120)	120 (90,150)	0.012	MD −16 (−29.3,−2.7)
Visual analogue scale (24 h), median (IQR)	2.0 (2.0,2.0)	2.0 (2.0,3.0)	0.128	MD 0.0 (−0.1,0.1)
Bleeding volume (mL), median (IQR)	2 (2,5)	2 (2,5)	0.377	MD 0.0 (−1.0,1.0)
Peritoneum or hernia sac tearing, *n* (%)				
No	40 (68.97)	37 (63.79)	0.694^a^	RR 0.86 (0.51,1.43)
Yes	18 (31.03)	21 (36.21)		
Hematoma, *n* (%)				
No	57 (98.28)	57 (98.28)	1.000^a^	RR 1.00 (0.06,15.61)
Yes	1 (1.72)	1 (1.72)		

a Fisher's exact test; MD: difference in medians (trans-arcuate line minus transumbilical), with 95% confidence interval; RR, risk ratio (trans-arcuate line versus transumbilical), with 95% confidence interval, calculated using the log-transformed (Katz) method; IQR, interquartile range.

**Table 7:** Analysis of key indicators in two groups of learning phase.

Characteristics	Trans-arcuate (*N* = 61)	Transumbilical (*N* = 16)	*p-*value	Effect estimate (95% CI)
Operative time (min), median (IQR)	120 (86,165)	115 (85,160)	0.683	MD 5.0 (−17.1,27.1)
Visual analogue scale (24 h), median (IQR)	2 (1,3)	2 (1,3)	0.233	MD 0.0 (−0.2,0.2)
Bleeding volume (mL), median (IQR)	3 (2,5)	2 (2,4)	0.547	MD 1.0 (−0.8,2.8)
Peritoneum or hernia sac tearing, *n* (%)				
No	44 (72.13)	6 (37.50)	0.017^a^	RR 0.45 (0.26, 0.78)
Yes	17 (27.87)	10 (62.50)		
Hematoma, *n* (%)				
No	61 (100.00)	16 (100.00)	Undefined	—
Yes	0 (0.00)	0 (0.00)		

a Fisher's exact test; MD: difference in medians (trans-arcuate line minus transumbilical), with 95% confidence interval; RR, risk ratio (trans-arcuate line versus transumbilical), with 95% confidence interval, calculated using the log-transformed (Katz) method; IQR, interquartile range.

## Discussion

Groin hernia, a prevalent surgical condition particularly affecting male patients, has a substantial impact on quality of life (1). In addition to causing localized pain and discomfort, it can lead to serious complications such as incarceration or strangulation of the hernial contents, thereby increasing the complexity of treatment and the associated healthcare burden (11, 12). With advancements in minimally invasive techniques, laparoscopic surgery has emerged as a prominent option for groin hernia repair, gaining widespread acceptance due to its minimal trauma and rapid recovery (13–15). However, traditional multi-port laparoscopic surgery is associated with drawbacks, including postoperative pain, extended recovery periods, and the risk of complications. Consequently, there has been a growing clinical interest in single-port laparoscopic techniques, which aim to achieve superior surgical outcomes and enhance patient experience (7, 9, 16).

This retrospective cohort analysis systematically evaluated the perioperative outcomes of trans-arcuate line and transumbilical approaches in TEP hernia repair. Phase-stratified CUSUM analysis revealed that the trans-arcuate line approach confers a lower peritoneum or hernia sac tearing rate during the learning phase and a shorter operative time after the learning curve is overcome, compared with the transumbilical approach in single-port TEP surgery. The differences in peritoneal injury observed in the overall matched cohort were primarily driven by the learning phase and did not persist after proficiency was reached. These findings suggest that the trans-arcuate line approach may be particularly beneficial for surgeons in the early stages of adopting single-port TEP surgery.

A particularly noteworthy finding emerged from the phase-stratified analysis of peritoneal injury. In the overall matched cohort, the peritoneum or hernia sac tearing rate was significantly lower in the trans-arcuate line group (30.1% vs. 47.0%, *p* = 0.026). However, after restricting the analysis to post-transition cases and re-matching, this difference was no longer statistically significant (31.0% vs. 36.2%, *p* = 0.694). Notably, the tearing rate in the trans-arcuate line group remained remarkably stable across phases (30.1% in the overall cohort, 31.0% in the post-transition phase, and 27.9% in the learning phase), whereas the transumbilical group exhibited a markedly higher tearing rate during the learning phase (62.5%) that declined substantially after proficiency was reached (36.2%). These findings suggest that the overall difference in peritoneal injury was primarily driven by the high tearing rate observed during the transumbilical learning phase, rather than by an inherent advantage of the trans-arcuate line approach that persists after proficiency. Rather, the trans-arcuate line approach may be more forgiving for surgeons in the early learning phase, as evidenced by its consistently low tearing rate irrespective of accumulated experience. Applying the same criterion used for operative time—which remained significant after re-matching, confirming a genuine advantage—the difference in peritoneal injury should be interpreted as phase-specific rather than persistent.

Neither recurrence nor length of hospital stay was assessed in this study, and neither is reported here. Patients in both cohorts were well-matched on key baseline characteristics after propensity score matching. This balance minimized the influence of known confounding factors on outcome measures, thereby supporting the attribution of observed differences primarily to the surgical approach itself. The cost of that balance is a narrower population: because every patient with an ASA score of III or IV was excluded by matching, the findings apply to an ASA I–II population only, as set out in the Limitations. After matching, 83 patients remained in each group. That number was determined by the pool of eligible cases available rather than by a power calculation; the confidence intervals in Table 5 are the appropriate guide to the precision achieved, and several secondary comparisons, that of haematoma among them, are too imprecise to be informative. The significant reduction in operative time underscores the efficiency advantage of the trans-arcuate line approach. The traditional transumbilical single-port approach requires instruments to traverse the narrow, potential space between the rectus abdominis muscle and the posterior sheath, and to cross the anatomical barrier of the arcuate line at depth before reaching the spacious preperitoneal operative field. This pathway not only presents initial spatial constraints and instrument interference (“chopstick effect”), but the arcuate line frequently obstructs the surgical view, increasing the difficulty and duration of anatomical dissection (9, 17). The trans-arcuate approach employed in this study (involving a longitudinal incision approximately 4 cm below the umbilicus) positions the incision directly at the level of the arcuate line. This allows instruments to bypass the aforementioned lengthy and narrow passageway, immediately accessing the lax region beneath the arcuate line upon entry. Consequently, a more direct and expansive operative field is achieved, significantly enhancing the efficiency of establishing the preperitoneal space (18–20). Reducing unnecessary tissue dissection and traction would also be expected to minimise local trauma and the accompanying inflammatory response. This is speculation: no marker of tissue trauma or inflammation was measured in this study, and any contribution of this mechanism cannot be separated from that of the shorter access route itself.

The “chopstick effect” refers to a phenomenon observed during endoscopic surgery where instruments entering the abdominal cavity from the same or adjacent entry points align almost parallel to each other, resembling chopsticks. This arrangement restricts the mobility of instrument tips within the surgical field, causes mutual interference, impedes the formation of an operational triangle, and compromises precise procedures such as dissection and suturing. The fundamental causes lie in the loss of the operational triangle and the lever effect. Ideal surgical operation requires the left and right instruments to converge at a specific angle (the optimal angle being 60°) to form a stable triangular configuration. As the incision is farther from the operative site, the angle between the instruments decreases and approaches parallel alignment, making manipulation more challenging; moving the incision closer to the target therefore improves triangulation—but only up to a point. That relationship is subject to a boundary condition. Moderate proximity widens the working angle towards the optimum of approximately 60°, because the instruments are brought nearer the field they must work in while still retaining room to splay; excessive proximity produces countervailing disadvantages, because the tips are then left too little room to manoeuvre, the working angle narrows again, and the configuration becomes unstable and has to be re-established repeatedly. Incision position and instrument angle are thus related in an inverted rather than a linear fashion, and there is an intermediate position at which triangulation is best. The trans-arcuate line approach is intended to place the incision nearer that intermediate position than the transumbilical route, which must traverse a longer, narrower channel and cross the arcuate line at depth before reaching the operative field. Whether it does so in practice was not measured here: no instrument angle, no assessment of instrument collision and no score of operative difficulty were recorded, and the argument is offered as a mechanistic explanation rather than as a finding of this study.

In single-port laparoscopic totally extraperitoneal groin hernia repair, the “chopstick effect” is plausibly an important mechanical factor in peritoneal injury, and the trans-arcuate line approach may reduce that risk by reshaping the instrument's operating triangle—subject to the boundary condition described above. The mechanism may involve three aspects. First, reduced instrument collision. In traditional transumbilical approaches, instruments and the camera occupy the single-port channel in parallel, with external handles colliding and internal tips forming a forceful lever; surgeons often unconsciously apply excessive force to overcome resistance. The trans-arcuate line approach places the instruments in a more V-shaped configuration, which we believe improves the lever arm ratio and reduces the risk of inertial bouncing or sliding perforation at the instrument tips. Second, with less instrument interference, the lens should be obstructed less often and the instrument's long axis should hold a more stable angle relative to the lens axis, which should help the surgeon to maintain depth control and to avoid peritoneal injury caused by manipulation in a blind spot. Finally, an optimised traction strategy: a better instrument angle should facilitate physiological reverse traction, converting destructive tearing forces into anatomically appropriate shear forces for separation. None of these mechanisms was measured in this study, and the explanation is accordingly speculative.

We consider single-port laparoscopic suturing of peritoneal defects to be the most challenging procedure, with significantly variable operative duration: successful cases may take 15–30 min, while complex or poorly positioned defects often require over 30 min or even necessitate conversion. A comparative study on the effects of defect closure vs. non-closure in laparoscopic totally extraperitoneal repair of direct inguinal hernias reported that the average operative time was 56.5 min in the unilateral hernia closure group vs. 45.7 min in the non-closure group, representing a difference of approximately 10.8 min (even for highly experienced surgeons), indicating that prolonged operative time is associated with additional procedures such as peritoneal defect management (21). Another study also identified independent risk factors for conversion during laparoscopic totally extraperitoneal inguinal hernioplasty, including large hernial defects and extensive peritoneal tears, all of which are closely related to difficulties in suturing the peritoneum (22). Therefore, the “trans-arcuate approach,” as a technical method for performing extraperitoneal suturing under direct vision, effectively addresses the challenges faced by novice and inexperienced surgeons in performing peritoneal sutures under laparoscopy or those requiring significant time expenditure, without the need for transposition or additional access ports, thereby offering notable clinical advantages. However, the trans-arcuate approach positions the incision closer to the operative field, which leads to a suboptimal instrument angle. This angle is unstable and requires frequent, extensive intraoperative adjustments, thereby compromising the fluidity of the surgical procedure (23).

The loss of statistical significance for VAS scores in the subgroup analysis may be partly attributable to the reduced sample size, and the potential benefit of the trans-arcuate line approach in reducing postoperative pain should not be definitively dismissed without further validation in larger cohorts. Similarly, the difference in peritoneal injury did not persist in the post-transition re-matched analysis; however, in contrast to VAS, the phase-specific pattern of peritoneal injury was more clearly delineated, with the overall difference being driven predominantly by the high tearing rate during the transumbilical learning phase. The advantages of the trans-arcuate approach in improving postoperative pain experience and preserving peritoneal integrity stem from the multifaceted benefits afforded by its optimised anatomical pathway. This approach avoids extensive dissection of the anterior plane of the rectus sheath, thereby reducing mechanical stimulation of abdominal wall nerve endings and potentially diminishing the release of inflammatory mediators. The shortened approach and reduced instrument interference enable the surgeon to more precisely navigate anatomical planes during dissection of the Retzius space, Bogros space, and hernial sac, thereby facilitating protection of neural, vascular, and spermatic cord structures. Should peritoneal damage occur, its proximity to the incision allows the surgeon flexible and expedient management via clamping, suturing, or other methods, avoiding the need for traumatic expansion or prolonged surgery due to repair difficulties. Preserving peritoneal integrity avoids the additional dissection, suturing and prolongation of surgery needed to repair a breach, and peritoneal injury is a recognised precursor of adhesion formation (24); measures that reduce peritoneal irritation have also been reported to lessen postoperative pain, although the mechanism is not established (25). The shorter operative time would also be expected to reduce anaesthetic exposure. No measure of anaesthetic dose or of systemic inflammatory response was collected in this study, so this remains a hypothesis rather than a finding.

It must be emphasised that this study did not assess recurrence. Follow-up was not standardised for the ascertainment of recurrence and the number of patients assessed at each time point could not be established reliably for either group, so no comparison of recurrence—and no claim about the durability of repair—can be offered here. Recurrence has been linked more closely to patient factors and to mesh characteristics than to the choice of approach alone (26, 27), but the present data cannot address that question, and the conclusions of this study are confined to the perioperative and early postoperative period. Furthermore, the central operative space afforded by the trans-arcuate approach is sufficiently spacious to facilitate bilateral groin hernia repair through the same incision, enhancing surgical efficiency.

This study has several limitations. First, the retrospective design introduces inherent selection bias, and the single-center nature of the data may limit the generalizability of the findings. Although propensity score matching was used to control for confounding, it cannot account for unmeasured confounders. The learning phase analysis was limited by the small sample size in the transumbilical group (*n* = 16), which precluded propensity score matching. Although standardized mean differences were generally small to moderate, some imbalances in sex, hernia laterality, and ASA grade were observed, and these unmeasured confounders may have influenced the observed difference in peritoneal injury. Therefore, the findings from the learning phase should be interpreted as hypothesis-generating rather than definitive Second, the two surgical approaches were not performed concurrently. The transumbilical approach was predominantly used from 2021 to 2022, while the trans-arcuate line approach became the main technique thereafter. Because a single surgeon performed every procedure, the relevant confounding is not diffuse across a team but is that surgeon's own experience curve, running in parallel with the change of technique. Differences in proficiency between the two periods may therefore have contributed to the results, and the point is taken further below. Third, follow-up was incomplete and was not standardised, and the evaluation focused on short-term perioperative outcomes. Recurrence, chronic pain and length of hospital stay were not assessed, and patients operated on in 2021 and in 2025 cannot be assumed to have had equivalent follow-up. Prospective studies with protocol-defined follow-up are warranted. Fourth, this study did not adopt the blinding method, and the surgical team could know the specific surgical approach used, which has a certain impact on the outcome assessment. Fifth, this study did not perform abdominal wall measurements, which, as an unmeasured confounding factor, may affect the assessment. We acknowledge that individualized landmark selection may be superior to fixed distance. Sixth, blood loss was visually estimated by the operating surgeon instead of formal volumetric measurement. We recognise that visual estimation of minimal bleeding has limited value for group comparison. Seventh, this study was not prospectively registered, and both the research protocol and the data collection process were completed under a retrospective framework, which may introduce methodological biases in multiple aspects. Eighth, the learning-curve analysis has limitations that bear directly on its interpretation. The CUSUM statistic is standardised within each group and necessarily returns to zero at the end of the series, so the two curves cannot be compared with one another and the descending limb cannot be read as evidence of a plateau; we have therefore taken the peak of each curve as the transition point. More importantly, the two approaches were practised in different periods by a single surgeon, so that surgeon's cumulative experience increased in parallel with the change of technique. Adjustment for chronological case number is the best correction available, cases beyond the transition phase from both groups were selected for re-comparison to clarify whether the observed differences persisted after the plateau phase. but it cannot fully separate the effect of the approach from the effect of accumulated experience, and the possibility that part of the difference in operative time—and in peritoneal breach, which is at least as likely to improve with experience—belongs to the learning curve rather than to the access route cannot be excluded. Ninth, the matched cohort comprised patients with an ASA score of I or II only. The trans-arcuate line group contained no ASA III or IV patients, the newer approach not having been offered to patients in these higher-risk categories during the study period; consequently all 20 such patients in the transumbilical group were excluded by matching. The findings therefore apply to an ASA I–II population and should not be extended to higher-risk patients, in whom the trans-arcuate line approach has not been evaluated. Tenth, follow-up was not standardised and neither the duration of follow-up nor the number of patients assessed at each visit was recorded consistently, so that recurrence, chronic pain and length of hospital stay are not reported and the conclusions are limited to the perioperative and early postoperative period.

In summary, this study demonstrates that in single-port TEP surgery, the trans-arcuate line approach offers a pathway design more aligned with local anatomy compared to the traditional transumbilical approach. After propensity score matching and phase-stratified analysis, the trans-arcuate line approach was associated with a lower peritoneum or hernia sac tearing rate during the learning phase, and with shorter operative time after proficiency was reached. These findings suggest that the trans-arcuate line approach may offer distinct advantages at different stages of the learning curve. No measure of operative difficulty was used and recurrence was not assessed, so no claim is made about either. On this evidence the trans-arcuate line approach is a reasonable alternative to the transumbilical route for single-port TEP groin hernia repair. The only safety outcome that favoured it was intraoperative peritoneal breach; haematoma, the other safety outcome recorded, did not differ significantly and was estimated very imprecisely, and conversion, wound complications, seroma and recurrence were not recorded. We therefore make no general claim that the trans-arcuate line approach is safer, and the findings require confirmation in a prospective study with protocol-defined follow-up.

## Data Availability

The original contributions presented in the study are included in the article/Supplementary Material, further inquiries can be directed to the corresponding author/s.
