# Supplementary material for: Trans-arcuate line vs. transumbilical access in single-port TEP groin hernia repair: a propensity score-matched retrospective cohort study
Source: Front Surg. 2026 Oct 13;13:1898654. doi: 10.3389/fsurg.2026.1898654 (PMC13710369; doi:10.3389/fsurg.2026.1898654)
Supplement: Supplementary file 2 [file Table2.docx]

Table 1. Baseline clinical data and patient characteristics before PSM

| Variable | Total (n = 292) | Trans-arcuate (n = 187) | Transumbilical (n = 105) | Statistic | *P* | SMD |
| --- | --- | --- | --- | --- | --- | --- |
|  |  |  |  |  |  |  |
| Age, Mean ± SD | 56.45 ± 16.54 | 55.27 ± 17.01 | 58.53 ± 15.52 | t=-1.621 | 0.106 | 0.210 |
| Sex, n (%) |  |  |  | χ²=0.047 | 0.828 |  |
| Male | 277 (94.86) | 177 (94.65) | 100 (95.24) |  |  | 0.028 |
| Female | 15 (5.14) | 10 (5.35) | 5 (4.76) |  |  | -0.028 |
| Hernia type, n (%) |  |  |  | - | 0.611 |  |
| Indirect inguinal hernia | 196 (67.12) | 124 (66.31) | 72 (68.57) |  |  | 0.049 |
| Direct inguinal hernia | 55 (18.84) | 39 (20.86) | 16 (15.24) |  |  | -0.156 |
| combined hernia | 36 (12.33) | 21 (11.23) | 15 (14.29) |  |  | 0.087 |
| Femoral hernia | 5 (1.71) | 3 (1.60) | 2 (1.90) |  |  | 0.022 |
| Hernia laterality, n (%) |  |  |  | χ²=2.965 | 0.227 |  |
| Left | 111 (38.01) | 69 (36.90) | 42 (40.00) |  |  | 0.063 |
| Right | 146 (50) | 91 (48.66) | 55 (52.38) |  |  | 0.074 |
| Bilateral | 35 (11.99) | 27 (14.44) | 8 (7.62) |  |  | -0.257 |
| History of abdominal surgery, n (%) |  |  |  | χ²=0.740 | 0.390 |  |
| no | 240 (82.19) | 151 (80.75) | 89 (84.76) |  |  | 0.112 |
| yes | 52 (17.81) | 36 (19.25) | 16 (15.24) |  |  | -0.112 |
| ASA score, n (%) |  |  |  | - | <.001 |  |
| I | 224 (76.71) | 162 (86.63) | 62 (59.05) |  |  | -0.561 |
| II | 48 (16.44) | 25 (13.37) | 23 (21.90) |  |  | 0.206 |
| III | 18 (6.16) | 0 (0.00) | 18 (17.14) |  |  | 0.455 |
| IV | 2 (0.68) | 0 (0.00) | 2 (1.90) |  |  | 0.139 |

Table 2. Baseline clinical data and patient characteristics after PSM

| Variable | Total (n = 166) | Trans-arcuate (n = 83) | Transumbilical (n = 83) | Statistic | *P* | SMD |
| --- | --- | --- | --- | --- | --- | --- |
|  |  |  |  |  |  |  |
| Age, Mean ± SD | 58.19 ± 16.15 | 58.69 ± 16.21 | 57.70 ± 16.17 | t=0.393 | 0.695 | -0.061 |
| Sex, n (%) |  |  |  | χ²=0.000 | 1.000 |  |
| Male | 156 (93.98) | 78 (93.98) | 78 (93.98) |  |  | 0.000 |
| Female | 10 (6.02) | 5 (6.02) | 5 (6.02) |  |  | 0.000 |
| Hernia type, n (%) |  |  |  | - | 0.943 |  |
| Indirect inguinal hernia | 116 (69.88) | 58 (69.88) | 58 (69.88) |  |  | 0.000 |
| Direct inguinal hernia | 28 (16.87) | 15 (18.07) | 13 (15.66) |  |  | -0.066 |
| combined hernia | 18 (10.84) | 8 (9.64) | 10 (12.05) |  |  | 0.074 |
| Femoral hernia | 4 (2.41) | 2 (2.41) | 2 (2.41) |  |  | 0.000 |
| Hernia laterality, n (%) |  |  |  | - | 0.935 |  |
| Left | 66 (39.76) | 34 (40.96) | 32 (38.55) |  |  | -0.050 |
| Right | 91 (54.82) | 45 (54.22) | 46 (55.42) |  |  | 0.024 |
| Bilateral | 9 (5.42) | 4 (4.82) | 5 (6.02) |  |  | 0.051 |
| History of abdominal surgery, n (%) |  |  |  | χ²=0.195 | 0.659 |  |
| no | 142 (85.54) | 72 (86.75) | 70 (84.34) |  |  | -0.066 |
| yes | 24 (14.46) | 11 (13.25) | 13 (15.66) |  |  | 0.066 |
| ASA score, n (%) |  |  |  | χ²=0.132 | 0.717 |  |
| I | 126 (75.9) | 64 (77.11) | 62 (74.70) |  |  | -0.055 |
| II | 40 (24.1) | 19 (22.89) | 21 (25.30) |  |  | 0.055 |

Table 3. Analysis of key indicators in different groups

| Characteristics | Trans-arcuate (N=83) | Transumbilical (N=83) | *p-*value | Effect estimate (95% CI) |
| --- | --- | --- | --- | --- |
| Operativetime(min), median (IQR) | 110 (93, 120) | 115 (97.5, 150) | 0.040 | MD = -5.0 (-9.6, -0.2) |
| Visual analogue scale, median (IQR) | 2 (2, 3) | 3 (2, 3) | < 0.001 | MD = -1.0 (-1.3, -0.7) |
| Bleeding volume (mL), median (IQR) | 2 (2, 5) | 2 (2, 5) | 0.420 | MD = 0.0 (-0.5, 0.7) |
| Peritoneum or hernia sac tearing, n (%) |  |  | 0.026^a^ | RR = 0.64 (0.42, 0.97) |
| No | 58 (69.9%) | 44 (53.0%) |  |  |
| Yes | 25 (30.1%) | 39 (47.0%) |  |  |
| Hematoma, n (%) |  |  | 0.367^b^ | RR = 4.01 (0.46, 35.1) |
| No | 79(95.2%) | 82 (98.8%) |  |  |
| Yes | 4 (4.8%) | 1 (1.2%) |  |  |

a: uncorrected Pearson chi-square; b: Fisher's exact test; MD: difference in medians (trans-arcuate line minus transumbilical), with 95% confidence interval; RR: risk ratio (trans-arcuate line versus transumbilical), with 95% confidence interval; IQR: interquartile range.

Table 4. Baseline clinical data and patient characteristics of post-transition and after PSM

| Variable | Total  (n = 116) | Trans-arcuate (n =58) | Transumbilical (n = 58) | Statistic | *P* | SMD |
| --- | --- | --- | --- | --- | --- | --- |
|  |  |  |  |  |  |  |
| Age, Mean ± SD | 58.22 ± 16.52 | 58.31 ±  16.67 | 58.14 ±  16.52 | t=0.056 | 0.955 | -0.010 |
| Sex, n (%) |  |  |  | χ²=0.000 | 1.000 |  |
| Male | 109 (93.97) | 55 (94.83) | 54 (93.10) |  |  | -0.068 |
| Female | 7 (6.03) | 3 (5.17) | 4 (6.90) |  |  | 0.068 |
| Hernia type, n (%) |  |  |  | - | 0.821 |  |
| Indirect inguinal hernia | 77 (66.38) | 39 (67.24) | 38 (65.52) |  |  | -0.036 |
| Direct inguinal hernia | 26 (22.41) | 14 (24.14) | 12 (20.69) |  |  | -0.085 |
| combined hernia | 11 (9.48) | 4 (6.90) | 7 (12.07) |  |  | 0.159 |
| Femoral hernia | 2 (1.72) | 1 (1.72) | 1 (1.72) |  |  | 0.000 |
| Hernia laterality, n (%) |  |  |  | - | 1.000 |  |
| Left | 39 (33.62) | 20 (34.48) | 19 (32.76) |  |  | -0.037 |
| Right | 69 (59.48) | 34 (58.62) | 35 (60.34) |  |  | 0.035 |
| Bilateral | 8 (6.9) | 4 (6.90) | 4 (6.90) |  |  | 0.000 |
| History of abdominal surgery, n (%) |  |  |  | χ²=0.058 | 0.809 |  |
| no | 95 (81.9) | 48 (82.76) | 47 (81.03) |  |  | -0.044 |
| yes | 21 (18.1) | 10 (17.24) | 11 (18.97) |  |  | 0.044 |
| ASA score, n (%) |  |  |  | χ²=0.000 | 1.000 |  |
| I | 94 (81.03) | 47 (81.03) | 47 (81.03) |  |  | 0.000 |
| II | 22 (18.97) | 11 (18.97) | 11 (18.97) |  |  | 0.000 |

Table 5. Analysis of key indicators in two groups of post-transition after PSM

| Characteristics | Trans-arcuate (N=58) | Transumbilical (N=58) | *p-*value | Effect estimate (95% CI) |
| --- | --- | --- | --- | --- |
| Operativetime(min), median (IQR) | 104 (83,120) | 120 (90,150) | 0.012 | MD -16 (-29.3,-2.7) |
| Visual analogue scale (24h), median (IQR) | 2.0 (2.0,2.0) | 2.0 (2.0,3.0) | 0.128 | MD 0.0 (-0.1,0.1) |
| Bleeding volume (mL), median (IQR) | 2 (2,5) | 2 (2,5) | 0.377 | MD 0.0 (-1.0,1.0) |
| Peritoneum or hernia sac tearing, n (%) |  |  |  |  |
| No | 40 (68.97) | 37 (63.79) | 0.694^a^ | RR 0.86 (0.51,1.43) |
| Yes | 18 (31.03) | 21 (36.21) |  |  |
| Hematoma, n (%) |  |  |  |  |
| No | 57 (98.28) | 57 (98.28) | 1.000^a^ | RR 1.00 (0.06,15.61) |
| Yes | 1 (1.72) | 1 (1.72) |  |  |

a: Fisher's exact test; MD: difference in medians (trans-arcuate line minus transumbilical), with 95% confidence interval; RR: risk ratio (trans-arcuate line versus transumbilical), with 95% confidence interval, calculated using the log-transformed (Katz) method; IQR: interquartile range

Table 6. Baseline clinical data and patient characteristics of learning phase

| Variable | Total  (n =77) | Trans-arcuate (n =61) | Transumbilical (n = 16) | Statistic | *P* | SMD |
| --- | --- | --- | --- | --- | --- | --- |
| Age, Mean ± SD | 56.65 ±  16.23 | 56.46 ±  16.42 | 57.38 ±  15.96 | t=-0.200 | 0.842 | 0.057 |
| Sex, n (%) |  |  |  | χ²=0.612 | 0.434 |  |
| Male | 71 (92.21) | 55 (90.16) | 16 (100.00) |  |  | 0.371 |
| Female | 6 (7.79) | 6 (9.84) | 0 (0.00) |  |  | -0.371 |
| Hernia type, n (%) |  |  |  | - | 0.930 |  |
| Indirect inguinal hernia | 51 (66.23) | 39 (63.93) | 12 (75.00) |  |  | 0.256 |
| Direct inguinal hernia | 14 (18.18) | 12 (19.67) | 2 (12.50) |  |  | -0.217 |
| combined hernia | 11 (14.29) | 9 (14.75) | 2 (12.50) |  |  | -0.068 |
| Femoral hernia | 1 (1.3) | 1 (1.64) | 0 (0.00) |  |  | -0.145 |
| Hernia laterality, n (%) |  |  |  | χ²=2.252 | 0.324 |  |
| Left | 27 (35.06) | 19 (31.15) | 8 (50.00) |  |  | 0.377 |
| Right | 40 (51.95) | 33 (54.10) | 7 (43.75) |  |  | -0.209 |
| Bilateral | 10 (12.99) | 9 (14.75) | 1 (6.25) |  |  | -0.351 |
| History of abdominal surgery, n (%) |  |  |  | χ²=0.000 | 1.000 |  |
| no | 66 (85.71) | 52 (85.25) | 14 (87.50) |  |  | 0.068 |
| yes | 11 (14.29) | 9 (14.75) | 2 (12.50) |  |  | -0.068 |
| ASA score, n (%) |  |  |  | χ²=0.326 | 0.568 |  |
| I | 61 (79.22) | 47 (77.05) | 14 (87.50) |  |  | 0.316 |
| II | 16 (20.78) | 14 (22.95) | 2 (12.50) |  |  | -0.316 |

Table 7. Analysis of key indicators in two groups of learning phase

| Characteristics | Trans-arcuate (N=61) | Transumbilical (N=16) | *p-*value | Effect estimate (95% CI) |
| --- | --- | --- | --- | --- |
| Operative time(min), median (IQR) | 120 (86,165) | 115 (85,160) | 0.683 | MD 5.0 (-17.1,27.1) |
| Visual analogue scale (24h), median (IQR) | 2 (1,3) | 2 (1,3) | 0.233 | MD 0.0 (-0.2,0.2) |
| Bleeding volume (mL), median (IQR) | 3 (2,5) | 2 (2,4) | 0.547 | MD 1.0 (-0.8,2.8) |
| Peritoneum or hernia sac tearing, n (%) |  |  |  |  |
| No | 44 (72.13) | 6 (37.50) | 0.017^a^ | RR 0.45  (0.26, 0.78) |
| Yes | 17 (27.87) | 10 (62.50) |  |  |
| Hematoma, n (%) |  |  |  |  |
| No | 61 (100.00) | 16 (100.00) | undefined | - |
| Yes | 0 (0.00) | 0 (0.00) |  |  |

a: Fisher's exact test; MD: difference in medians (trans-arcuate line minus transumbilical), with 95% confidence interval; RR: risk ratio (trans-arcuate line versus transumbilical), with 95% confidence interval,calculated using the log-transformed (Katz) method; IQR: interquartile rang
